# Psychological and Clinical Parameters as Predictors of Relapse in Alcohol-Dependent Patients During and After Extensive Inpatient Rehabilitation Treatment

**DOI:** 10.3390/brainsci15040374

**Published:** 2025-04-03

**Authors:** Josef Rabl, Dieter Geyer, Katharina Steiner, Fabrizio Schifano, Norbert Scherbaum

**Affiliations:** 1 LVR-University Hospital Essen, Department of Psychiatry and Psychotherapy, Faculty of Medicine, University of Duisburg-Essen, Virchowstr. 174, 45147 Essen, Germany; josef.rabl@stud.uni-due.de (J.R.);; 2 Johannesbad Kliniken Fredeburg GmbH, Zu den Drei Buchen 1, 57392 Schmallenberg, Germany; 3 Psychopharmacology, Drug Misuse and Novel Psychoactive Substances Research Unit, School of Life and Medical Sciences, University of Hertfordshire, College Lane Campus Hatfield, Hatfield AL10 9AB, UK; f.schifano@herts.ac.uk

**Keywords:** alcohol use disorder, alcohol dependence, craving, attentional bias, impulsivity, inhibitory control, relapse

## Abstract

Background: Psychological parameters related to alcohol dependence (AD) affect patients’ behavioral and cognitive control, decision making, impulsivity and inhibitory control. People with AD often have a chronic course with a relapse to dependent substance use even after extensive treatment. This study investigated whether the psychological parameters of patients with AD predict (a) premature termination of treatment, and/or (b) relapse into consumption of alcohol from admission until 6 weeks after discharge from an inpatient rehabilitation treatment. Methods: Participants: Alcohol-dependent patients consecutively admitted for a duration of about three months to inpatient rehabilitation treatment in a hospital specialized in substance use disorders. Craving (OCDS-G) and impulsivity (BIS-11; UPPS) were assessed with computerized questionnaires. Attentional bias and inhibitory control were measured with two computer-based experiments (dot-probe task; stop-signal task (SST)). Investigations were conducted at entry (T1); after 6 weeks (T2); and during the last two weeks of the inpatient treatment (T3). Some *N* = 128 patients finished the first, *N* = 102 the second and *N* = 83 the third assessments. Outcome variables were discontinuation of treatment and abstinence or relapse until follow-up 6 weeks after discharge; participants were contacted via telephone. Results: None of the variables are associated with discontinuation of treatment. Poor inhibitory control (SST) and high craving (OCDS-5) levels, measured at T1, are significantly associated with relapse. Higher impulsivity (UPPS) measured at T2 and T3 is significantly associated with relapse. Exploratory analyses showed that older age, longer inpatient treatment duration and time spent in abstinence before rehabilitation treatment were significantly associated with a reduced risk of relapse. Conclusions: Psychological parameters, craving and impulsivity levels did not predict relapse to a high degree. It is assumed that discontinuation of treatment and relapse may be associated with different issues, such as social context, and individual motivation levels. In contrast, the length of both abstinence before admission and of inpatient treatment were significantly associated with abstinence; it is here suggested that recovery time duration may be an underestimated influencing factor regarding relapse in AD patients.

## 1. Introduction

Alcohol dependence (AD) is a psychiatric disorder that is both highly prevalent in western countries and associated with substantial psychological and physiological health damage, including morbidity, mortality and psychosocial impairments [1,2,3]. Genetic factors influence the etiology and course of AD [4]; the current research identified a polygenic effect on AD treatment outcomes, such as relapse [5]. AD is further associated with changes regarding several psychological parameters [6,7,8]. Psychological parameters in AD patients relate to diminished response inhibition [9], diminished overall cognitive performance and impaired working memory levels [10,11]. In addition, similar to other substance-related dependencies, AD is associated with high levels of impulsivity [12,13], an attentional bias toward substance-related cues [14,15,16], decreased inhibitory control [17,18,19] and substance craving [20,21,22].

Furthermore, alcohol craving [20,23,24,25], attentional bias [26,27], impulsivity [28,29] and poor inhibitory control [30,31,32] are predictors of relapse. However, most studies evaluated only one or two variables, and in particular investigated samples of patients in detoxification treatment units from the USA, where the duration of detoxification treatment varies between 3 to 14 days [33,34]. In addition, few patients in these studies engage in follow-up treatment after detoxification [35]. Therefore, it can be debated whether those patients were provided with sufficient levels of AD treatment.

Overall, there is a lack of studies investigating the predictive value of psychological parameters after extensive treatment, e.g., carried out over several months. The inpatient rehabilitation treatment in Germany offers the possibility to evaluate the predictive value of these parameters in patients with AD. Treatment duration for AD is around three months. Inpatient rehabilitation treatment is widely available in Germany, financed either by pension funds or, in the minority of cases, by the statutory health insurances. Prior to admission to a rehabilitation treatment center, patients usually undergo inpatient detoxification treatment lasting up to 21 days.

The inpatient rehabilitation treatment package consists of a variety of treatment elements, including weekly individual psychotherapy sessions, focusing, e.g., on craving management, better regulation of negative emotions and reducing impulsivity; group psychotherapy sessions several times a week; occupational assessment and therapy; career and social counseling; sport therapy and support to organize a follow-up treatment, facilitating a connection with self-help groups. However, follow-up treatment did not take place in the hospital, where the inpatient treatment took place. There are no data regarding the question of whether patients actually started the planned follow-up treatment. During inpatient treatment, alcohol abstinence is confirmed through regular unannounced breathalyzer tests and urine samples (e.g., identification of ethyl glucuronide). After discharge, follow-up treatment is offered in specialized institutions for up to an additional 40 weeks. In an assessment one year after the discontinuation or regular termination of inpatient rehabilitation treatment, relapse rates varied greatly, for example between 61.5% in the assessment’s total sample (all patients discharged in 2018, *N* = 4.365), and 22.2% for a subsample only including patients who completed their treatment regularly and responded to the catamnestic survey (*N* = 2.031) [36].

Hence, we aimed here to assess if either a battery of psychological functions or a range of remaining factors would be associated with the discontinuation of inpatient rehabilitation treatment and/or with relapse during inpatient treatment and up to a follow-up six weeks after discharge from the hospital. In addition, we aimed at an exploratory analysis of the predictive value of the clinical data (age, sex, psychiatric and substance-related comorbidities, duration of time spent in abstinence before admission, duration of treatment) regarding therapy discontinuation and relapse.

## 2. Materials and Methods

### 2.1. Study Design, Setting, and Participants

This longitudinal, observational study was conducted in the Johannesbad Fachklinik Fredeburg (FKF) clinic in Germany, specialized in the inpatient rehabilitation treatment of patients with AD. Participants were recruited between the 4 July 2022 and the 15 April 2023. Data collection ended on the 23 August 2023. Eligibility criteria included: diagnosis of alcohol dependence according to ICD-10; absence of an unstable schizophrenic disorder or epilepsy; and a maximum age of up to 65 years. Comorbid mental disorders were documented for exploratory analyses. Possibly eligible patients were asked to participate in the study. Patients were informed about the study aims, procedures and the possible risks and benefits of this study. After signing the consent form, participants were assigned to the first examination and were informed as well in terms of dates of further examinations via their weekly schedule provided by the clinic; all participants received the hospital’s usual treatment package.

This study consisted of three examinations, which were conducted during the first week of treatment, six weeks into it and within the last two weeks of treatment. To evaluate the association of psychological parameters with a possible eventual relapse, patients were asked about their abstinence by phone six weeks after discharge from inpatient treatment. At the follow-up assessment, patients were asked on the phone: “Since your discharge from the clinic, did you consume any alcohol or drugs other than nicotine”? Any consumption of alcohol or other illicit drugs was classified as relapse.

All examinations were carried out with the aid of a 19-inch screen with a 4:3 aspect ratio. Participants used two keys on a German computer keyboard to complete the related psychological tasks and a mouse to answer the questionnaires. Inquisit Lab 6, version 6.6.1 [37] was used to execute the two reaction-time experiments and the processing of the questionnaires. If not provided by Inquisit Lab 6, version 6.6.1 [37], the experiments’ instructions were translated into German language.

### 2.2. Variables

Putative predictors of premature treatment discontinuation and relapse at the 6-week follow-up included: alcohol craving, impulsivity, inhibitory control and attentional bias toward alcohol related cues, alongside a range of possible confounders, such as comorbidities regarding another substance-related disorder or other psychological disorders, and demographic data.

#### 2.2.1. Alcohol Craving

The short version of the Obsessive Compulsive Craving Scale (German Version) (OCDS-G), which is known for its economic use and proven reliability, was chosen to measure the patients’ cravings [38,39]. To prevent the following experiments from influencing the participants’ cravings, the questionnaire was placed first in the examination.

#### 2.2.2. Attentional Bias

Positioned second was the alcohol dot-probe experiment [40,41]. Following a fixation cross, two images appeared side by side for a duration of 1000 ms. They showed either non-alcoholic/alcoholic beverages or random objects. Shortly afterward, a probe (an “X”) was presented on the same position as one of the pictures. Participants were instructed to press one of two assigned keys as fast as possible to indicate the probe’s location. Trials were counted only if one of the keys was pressed within 1000 ms. An attentional bias toward alcohol was assessed by calculating the reaction time in alcohol congruent trials versus non-congruent trials. A positive deviation (faster reaction time in alcohol congruent trials) indicated an attentional bias. High-resolution pictures of German alcoholic beverages replaced the original stimuli of beverages from North America, to ensure that the participants from Germany were able to accurately differentiate between alcoholic and non-alcoholic beverages.

#### 2.2.3. Impulsivity

The German versions of the UPPS Impulsive Behavior Scale (UPPS) [40,42,43] and the Baratt Impulsiveness Scale (BIS-11) [44,45,46] were digitalized with Inquisit Lab 6, version 6.6.1 [37] and then used to assess the participants’ impulsivity levels.

#### 2.2.4. Inhibitory Control

The stop-signal task (SST), measuring inhibitory control via an estimated stop-signal reaction time, was used. It implemented the recommendations of the consensus guide by Verbruggen et al. to attain the optimal validity of the stop-signal reaction time [47,48]. A fixation circle was presented and soon after an arrow appeared inside the circle, randomly pointing left or right. Participants were instructed to indicate the arrows’ direction as fast and precise as possible using two keys on the computers keyboard. After the arrow appeared, a short beep was played via the computer’s speakers at random time points. The delay of the beep started at 250 ms and was adjusted in 50 ms increments up or down automatically by the software in relation to the participants’ performance. Participants were instructed to inhibit their reaction when this stop-signal appeared. The signals’ volume was set to a comfortable level with the participants. Datasets were analyzed according to the methodological paper of Verbruggen et al. (2019) [48].

#### 2.2.5. Treatment Discontinuation and Abstinence vs. Relapse

The dependent variables of this study included the participants’ discontinuation of treatment and their abstinence from alcohol and other drugs (other than tobacco) up to the 6-week post-discharge follow-up. Information regarding participants’ discontinuation of treatment was available from the clinical database of the hospital. To assess abstinence vs. relapse, participants were contacted via phone 6 weeks after their discharge and were asked if they had consumed any alcohol or other psychotropic drugs, since most participants presented with remaining SUDs alongside their AD, after their discharge from the clinic. The consumption of alcohol or other psychotropic substances other than nicotine was categorized as a relapse, regardless of the amount and duration of consumption. If participants could not be reached, further calls were made over the following two weeks. If participants could repeatedly not be reached on at least five consecutive occasions, they were conservatively categorized as relapsers. Participants suffering a relapse during their inpatient treatment were classified as relapsers as well.

#### 2.2.6. Exploratory Variables

Several clinical variables that are mostly part of the hospital’s routine dataset were exploratory analyzed regarding their prediction of discontinuation of inpatient treatment and/or within- and post-treatment relapse.

##### Time Spent in Abstinence Before Admission and During Treatment

The number of days spent in abstinence before admission was reported by the patients at the initial study assessment. The duration of treatment was provided by the hospital’s routine data.

##### Demographic Data and Comorbid Psychiatric and Substance-Related Disorders

The variables age, sex, number of substance-related disorders and other psychiatric diagnoses were provided by the hospital’s routine data.

### 2.3. Statistical Methods

Statistical analyses were calculated using software R, version 4.2.2 [49], with the graphical user interface RStudio, version 2023.06.0, build 421 [50]. The participants’ craving, attentional bias, impulsivity and inhibitory control were evaluated for significant effects on the participant’s discontinuation of treatment and abstinence or relapse within the timespan of treatment and until 6 weeks after the end of their treatment, using logistic regression models.

## 3. Results

### 3.1. Descriptive Statistics

#### 3.1.1. Recruitment Success, Dropouts, and Final Sample

A total of *N* = 172 patients were asked to participate, and *N* = 152 agreed to be included in this study. Out of these, *N* = 19 did not complete the first examination due to reasons such as the need of an urgent medical intervention; identification of formerly unknown diagnoses, which violated the inclusion criteria; cognitive limitations preventing the understanding of the study tasks or discontinuation of treatment prior to starting the first examination. In addition, *N* = 3 patients did not attend the assessment appointments and, hence, were excluded from the study, and *N* = 2 participants were excluded, due to either their examination not having been fully carried out or a computer problem causing the total loss of data. Hence, *N* = 128 successfully completed the first assessment, *N* = 102 completed the second and *N* = 83 completed the third assessment. Reasons for dropouts included: discontinuation of the inpatient treatment (*N* = 30); refusal to continue participating to the study (*N* = 6); relapse into alcohol use whilst still hospitalized (*N* = 2) and others (*N* = 7).

#### 3.1.2. Demographic Data

One hundred participants were males and 28 females; mean age was 43.30 years (*SD* = 11.09; range: 18–64 years). Regarding their schooling levels, *N* = 27 (21.1%) had completed high school, *N* = 78 (60.9%) had completed either vocational training or middle school and *N* = 6 (4.7%) had obtained a university diploma (for more details, see Table 1).

#### 3.1.3. Comorbid Psychiatric and Substance-Related Disorders

*N* = 97 (75.8%) of participants were diagnosed with nicotine addiction (ICD-10 F17.2); *N* = 62 (48.4%) suffered from another SUD and *N* = 40 (31.3%) suffered from 2 or more SUD diagnoses (see Table 1). Comorbid mental disorders were identified in *N* = 65 (50.8%) of the sample (see Table 2).

#### 3.1.4. Time Spent in Abstinence Before Admission and Within Treatment

On average, participants remained in the clinic for 103.39 days (*SD* = 27.48) until the third assessment. Mean duration of alcohol abstinence prior to admission was 34.72 days (*SD* = 44.37).

#### 3.1.5. Treatment Discontinuation and Within-Treatment Relapse

Of the 128 participants, a total of *N* = 30 (23.4%) prematurely discontinued the inpatient treatment. Of those *N* = 30, a total of *N* = 6 suffered from relapse in the clinic, before their subsequent discontinuation of treatment. Another *N* = 3 patients suffered from relapse during treatment after the completion of the second assessment but completed therapy after their relapse; they hence were not included in the third assessment. The *N* = 9 during-treatment relapsers were excluded from the follow-up survey assessing post-treatment relapse. The relapse rate of the complete sample (*N* = 128), including during-treatment relapse, was 17.96% (*N* = 23) (see Table 3).

#### 3.1.6. Post-Treatment Relapse

Regarding the follow-up assessment for post-treatment relapse (*N* = 119), a total of *N* = 80 or 67.2% of the participants could be successfully contacted; out of these, *N* = 66 or 82.5% reported that they had been able to maintain full sobriety in the post-discharge period; while *N* = 14 or 17.5% had allegedly relapsed and *N* = 39 participants (30.5%) could not be contacted (see Table 3).

#### 3.1.7. Means and Standard Deviations of the Variables

The variables’ means and standard deviations differed across the timepoints of the assessment (see Table 4). For more information on the progress of the variables over the duration of treatment, see our article [51].

### 3.2. Statistical Analyses

#### 3.2.1. Statistical Analyses of the Variables Regarding Treatment Discontinuation

No overall significant statistical associations were found here between craving; attentional bias; impulsivity and inhibitory control (e.g., as measured at both admission and 6 weeks later) and rates of treatment discontinuation. Of the exploratory variables, older age was associated significantly with less rates of treatment discontinuation (see Table 5).

#### 3.2.2. Statistical Analyses of the Variables Regarding Relapse

Conversely, the logistic regression analysis showed that the first examination (e.g., at admission) OCDS-5 craving scores were significantly associated with both the observed levels of relapse and the SST scores. The explorative variables age, days abstinent before admission and duration of treatment all were significantly associated with relapse (see Table 6).

This may suggest that both high levels of craving and poor SST reaction times increased the eventual patients’ relapse risk. Furthermore, the UPPS impulsivity scores measured at both 6 weeks of inpatient treatment (*N* = 102) and shortly before discharge (*N* = 83) were significantly associated with the relapsing levels (see Table 6).

#### 3.2.3. Comparison of the Logistic Regression Models

Concerning the further range of exploratory variables here assessed for the whole sample (*N* = 128), a negative significant association was identified between participants’ age and discontinuation of inpatient treatment (see Table 5). Furthermore, the relapsing levels were negatively associated with both the number of abstinent days prior to the clinic admission and duration of inpatient treatment in days (see Table 6). Hence, overall, those who were older, presenting with a longer period of alcohol abstinence before admission and with a longer treatment duration were less likely to discontinuing the inpatient treatment or suffering from an alcohol relapse. The exploratory logistic regression models presented better fit indices regarding therapy discontinuation and relapse than the models containing the experimental variables as predictors, with a Nagelkerke *R*^2^ of 0.41 (see Table 7).

## 4. Discussion

Previous studies demonstrated that alcohol craving [20,23,24,25], attentional bias [26,27], impulsivity [28,29] and poor inhibitory control [30,31,32] as empirically proven psychological predictors of relapse in persons with AD. These findings could have clinical implications as these psychological parameters could be influenced by specific treatment approaches to improve the further course of alcohol dependence. The current study was carried out to assess whether these psychological parameters, measured over the course of an inpatient rehabilitation treatment, could predict both discontinuation of inpatient treatment and/or relapsing rates at the 6-week post-discharge follow-up. No significant associations were here identified between the variables of interest and the discontinuation of treatment. Conversely, a significant association between the craving levels at admission, but not at both the 2nd and 3rd examinations, and the relapse risk at follow-up was found here. The predictive value of craving at treatment admission could help the therapeutic team to address the issue of substance craving with adequate psychological and medical interventions and, from the very beginning, to prevent early treatment discontinuation. In the following weeks, the alcohol craving levels decreased in most patients, hence confirming the previous suggestions [51]. This was possibly due to the positive effects of the clinic secure environment, characterized by lack of access to alcohol/drugs; provision of arrange of daily therapeutic interventions; not meeting with dealers/problematic acquaintances and the prompt availability of health personnel. All these factors may well have contributed to the decrease in craving levels observed.

The SST investigated the ability to inhibit planned actions. Rejecting the offer of an alcoholic beverage may be seen as an example of a successful inhibition in patients’ daily lives. SSRT values (e.g., measuring inhibitory control) at admission, but not afterward, showed a significant association with the occurrence of a later relapse. SSRT values were not however associated with early therapy discontinuation rates, regardless of the timepoint of examination. The current results are at odds with previous studies investigating the impact of response inhibition on therapy discontinuation and relapse in AD patients [31,52], although variations in the methodology could explain such differences in the predictive value of SSRT itself [48].

In the current study, UPPS-related impulsivity levels at admission were not significantly associated with the subsequent relapsing levels here observed. Conversely, UPPS values, as measured at the 2nd and 3rd assessments, were significantly related to a relapse risk afterward. Impulsivity levels at admission were however high for most patients, hence this parameter was unable to provide any discriminating predictive value. Conversely, impulsivity scores remained high over the whole course of treatment for only some patients and this was associated with a significantly high relapse risk at follow-up. Indeed, one could argue that in the post-discharge period, when exposure to alcohol-related cues is frequent, the chances of successfully coping with alcohol cravings may be optimal only when the individual impulsivity levels are minimal.

In contrast to UPPS, the BIS-11 impulsivity self-report questionnaire did not show any significant association with relapse at all three examination times. The BIS-11 was however developed for use in young adults, and the mean age of the current sample (e.g., 43.3 years) could have reduced the predictive validity of BIS-11 itself.

The attentional bias toward alcohol-related stimuli, here measured by the dot-probe task, did not show any significant association with relapsing rates at follow-up. The current findings confirm previous observations from our group [51], but are in contrast with those previous suggestions indicating an association between attentional bias levels and treatment outcomes in AD patients [53,54,55].

Out of all the exploratory variables here examined, only participants’ age was significantly associated with levels of risk of treatment discontinuation, hence confirming the previous research observations [56]. Furthermore, older age, a higher number of abstinent days prior to admission and longer treatment duration were all associated with smaller levels of relapsing risks. Indeed, the ability of a patient to maintain abstinence for some time before admission may reflect both his/her motivation to change and the existence of an already progressed recovery prior to admission [57]. The current data show that, similar to what was observed in a dosage–effect curve, the longer the patients are treated, the less likely they suffer from a post-discharge relapse. The exploratory logistic regression models demonstrated better fit indices when compared to the models incorporating the experimental data in T1–T3 as predictors, raising the question of whether the predictive value of the exploratory data exceeds that of the experimental variables (see Table 7).

Former studies reporting craving attentional bias, impulsivity and inhibitory control as psychological parameters to predict relapse in AD patients suggest that such psychological parameters should be influenced by specific treatment approaches to improve the prognosis of alcohol dependence. However, our data did not confirm the results of previous studies carried out in other settings. Hence, our results can allow us to critically discuss the focus on control of alcohol craving, cognitive bias modification, impulsivity and inhibitory control as elements of rehabilitation treatment.

It has to be stressed here that relapsing after long-term inpatient treatment might be caused by a variety of factors and not only by the psychological parameters investigated here. Indeed, patients may have differed from each other here in terms of their social situation, e.g., being employed vs unemployed; being in a supportive relationship or not; being socially isolated or not, etc. These factors might even have interacted here with psychological parameters increasing or decreasing their effects. However, the analysis of post-discharge-related social factors was beyond the scope of this investigation. There was no significant association with the participants’ sex and therapy discontinuation and relapse, which could be explained by the unbalanced distribution of sex across the sample, leading to a smaller sample size of female participants.

Whilst alcohol abstinence was almost secured here due to both stringent clinic regulations and random inspections, it could not be completely guaranteed. Again, due to limited financial resources, advanced technology, such as eye-tracking measures, could not be considered. Furthermore, to confirm the study participants’ abstinence status at follow-up, we relied on individual allegations. Finally, the current sample consisted of patients suffering not only from AD but also from other SUDs, and this may have complicated the interpretation of the present findings.

As usual, in clinical samples of persons with AD, the majority of patients suffer from comorbid substance-related disorders. Only 26 patients (20.3%) were (apart from nicotine addiction) diagnosed with AD only (see Table 1). Hence, one could argue that an investigation of persons who are only dependent on alcohol might have had a different result. However, it has to be stressed that the number of comorbid substance-related disorders and/or psychiatric disorders did not show a significant association with either therapy discontinuation or relapse. In addition, this study was performed on a sample representative for usual clinical samples in the treatment of alcohol dependence. This study’s relatively low sample size is another potential limitation. Nevertheless, it is noteworthy that the current research suggests the potential for the identification of a substantial effect of the variables in question, despite the limitation of the sample size [58]. The age of onset of substance use was proven to be a significant factor associated with the severity of cognitive deficits in polyconsumer men [59]. However, the age of onset of alcohol and substance use was not recorded in this study. The use of highly sophisticated instruments (e.g., eye tracking) for the assessment of attentional bias in substance-use disorders [60] could have helped here in providing a more precise determination of bias, hence possibly contributing to a better prediction of relapse.

## 5. Conclusions

Psychological parameters, including craving and impulsivity levels, were unhelpful as predictors of abstinence at follow-up for patients with alcohol dependence six weeks after discharge from inpatient rehabilitation treatment. In contrast, duration of abstinence before admission and length of the inpatient treatment duration were significantly associated with abstinence. One could argue that the length of the recovery time may be an underestimated factor influencing post-discharge alcohol abstinence.

## Figures and Tables

**Table 1:** Demographic data of the total sample, *N* = 128.

	*M* (*SD*) Frequency (%) Min/Max
Age	43.30 (11.09) 18/64
Female/male	28 (21.9%)/100 (78.1%)
Educational background	
No degree	6 (4.7%)
Basic degree	11 (8.6%)
Secondary school (10th class or vocational training)	78 (60.9%)
High school	27 (21.1%)
University diploma	6 (4.7%)
Number of SUD diagnoses	
1	26 (20.3%)
2	62 (48.4%)
3 or more	40 (31.3%)
Number of psychiatric diagnoses	
0	63 (49.2%)
1	50 (39.1%)
2 or more	15 (11.7%)

*M* = mean value, *SD* = standard deviation, Min = minimum, and Max = maximum.

**Table 2:** Additional substance-related and categories of psychiatric diagnoses according to ICD-10.

	Frequency (%)
Comorbid mental and behavioral disorders due to use of:	
F17.2 Tobacco	97 (75.8)
F11.2 Opioids	1 (0.8)
F12.2 Cannabinoids	25 (19.5)
F13.2 Sedatives or hypnotics	3 (2.3)
F14.2 Cocaine	11 (8.5)
F15.2 Other stimulants, including caffeine	13 (10.0)
Other psychiatric diagnoses:	
F3 Mood [affective] disorders	43 (33.5)
F4 Neurotic, stress-related and somatoform disorders	22 (17.1)
F5 Behavioural syndromes associated with physiological disturbances and physical factors	1 (0.8)
F6 Disorders of adult personality and behaviour	14 (10.9)
F9 Behavioural and emotional disorders with onset usually occurring in childhood and adolescence	6 (4.6)

**Table 3:** Results of the follow-up examination.

	Status at Follow-Up *N* (%)		
Group	Abstinence	Relapse	Not Reachable
Completers of the first assessment, including during-treatment and post-treatment relapse (*N* = 128)	66 (51.56)	23 (17.96)	39 (30.46)
Completers of the first assessment, including post-treatment relapse (*N* = 119)	66 (55.46)	14 (11.76)	39 (32.77)
Completers of the second assessment, only post-treatment relapse (*N* = 97)	62 (63.92)	10 (10.31)	25 (25.77)
Completers of the third assessment, only post-treatment relapse (*N* = 83)	53 (63.85)	8 (9.63)	22 (26.50)

**Table 4:** Values of the variables grouped by timepoint of assessment.

	T1 *M*, (*SD*)	T2 *M*, (*SD*)	T3 *M*, (*SD*)
Craving: OCDS-5	5.29 (3.60)	2.93 (2.49)	2.23 (2.14)
Attentional Bias: Dot Probe	1.48 (26.52)	−3.99 (24.75)	0.74 (19.46)
Impulsivity: BIS-11	67.37 (10.73)	66.35 (10.61)	65.02 (10.86)
Impulsivity: UPPS	109.84 (16.67)	107.76 (14.84)	105.48 (16.01)
Inhibitory Control: SST	221.14 (60.19)	220.16 (51.24)	224.30 (37.33)

T1–3 = timepoint, *M* = mean, and *SD* = standard deviation, rounded to two digits.

**Table 5:** Predictive values of the variables regarding therapy discontinuation.

	Estimate	Std. Error	z Value	*p*	*OR*
Predictive value of the variables at timepoint/assessment 1 regarding therapy discontinuation, *N* = 128.					
Intercept	−3.124553	1.706484	−1.831	0.0671	
Craving	0.006889	0.065129	0.106	0.9158	1.01
Attentional Bias	−0.009251	0.008790	−1.052	0.2926	0.99
BIS-11	−0.013818	0.031142	−0.444	0.6572	0.99
UPPS-G	0.017079	0.021566	0.792	0.4284	1.02
Stop-Signal Task	0.004203	0.003526	1.192	0.2333	1.00
Predictive value of the variables at timepoint/assessment 2 regarding therapy discontinuation, *N* = 102.					
Intercept	−6.288740	3.949806	−1.592	0.111	
Craving	0.020909	0.167381	0.125	0.901	1.02
Attentional Bias	0.020890	0.022260	0.938	0.348	1.02
BIS-11	−0.011213	0.057251	−0.196	0.845	0.99
UPPS-G	0.015931	0.041722	0.382	0.703	1.02
Stop-Signal Task	0.011438	0.009009	1.270	0.204	1.01
Predictive value of the exploratory variables regarding therapy discontinuation, *N* = 128.					
Intercept	0.633038	1.168447	0.542	0.588	
Age	−0.052865	0.022356	−2.365	0.018 *	0.95
Sex	−0.249035	0.589497	−0.422	0.673	0.78
Days Abstinent Before Admission	−0.002746	0.005994	−0.458	0.647	0.99
Number of Psychiatric Diagnoses	−0.165511	0.283701	−0.583	0.560	0.85
Number of SUD Diagnoses	0.263094	0.233488	1.127	0.260	1.30

Logistic regression models, dependent variable = therapy discontinuation 0/1; * = *p*-value < 0.05, *OR* = Odds Ratio, rounded to 2 digits.

**Table 6:** Predictive values of the variables regarding relapse.

	Estimate	Std. Error	z Value	*p*	*OR*
Predictive value of the variables at timepoint/assessment 1 regarding relapse, *N* = 128.					
Intercept	−3.3533168	1.5473027	−2.167	0.0302 *	
Craving	0.1536928	0.0635274	2.419	0.0155 *	1.17
Attentional Bias	−0.0072125	0.0075551	−0.955	0.3398	0.99
BIS-11	0.0008643	0.0280941	0.031	0.9755	1.00
UPPS-G	0.0089188	0.0193042	0.462	0.6441	1.01
Stop-Signal Task	0.00659993	0.0033529	0.462	0.0490 *	1.01
Predictive value of the variables at timepoint/assessment 2 regarding relapse, *N* = 102.					
Intercept	−4.571574	1.953678	−2.340	0.0193 *	
Craving	0.101813	0.091592	1.112	0.2663	1.11
Attentional Bias	0.013867	0.010966	1.265	0.2060	1.01
BIS-11	−0.022067	0.030778	−0.717	0.4734	0.98
UPPS-G	0.052914	0.023866	2.217	0.0266 *	1.05
Stop-Signal Task	−0.001912	0.004229	−0.452	0.6512	1.00
Predictive value of the variables at timepoint/assessment 3 regarding relapse, *N* = 83.					
Intercept	−3.431949	2.470477	−1.389	0.1648	
Craving	0.022323	0.116806	0.191	0.8484	1.02
Attentional Bias	−0.012388	0.012510	−0.990	0.3221	0.99
BIS-11	−0.028235	0.032183	−0.877	0.3803	0.97
UPPS-G	0.046821	0.023702	1.975	0.0482 *	1.05
Stop-Signal Task	−0.001483	0.006391	−0.232	0.8165	1.00
Predictive value of the exploratory variables regarding relapse, *N* = 128.					
Intercept	3.467787	1.329495	2.608	0.00910 **	
Age	−0.053196	0.021509	−2.473	0.01339 *	0.95
Sex	−0.577035	0.551916	−1.046	0.29579	0.56
Days Abstinent Before admission	−0.021608	0.007373	−2.931	0.00338 **	0.98
Duration of Treatment (Days)	−0.018755	0.006062	−3.094	0.00198 **	0.98
Number of Psychiatric Diagnoses	0.636354	0.327422	1.944	0.05195	1.89
Number of SUD Diagnoses	0.345315	0.257891	1.339	0.18057	1.41

Logistic regression models, dependent variable = relapse 0/1; * = *p*-value < 0.05, ** = *p*-value < 0.01, *OR* = Odds Ratio, rounded to 2 digits.

**Table 7:** Model fit parameters of the logistic regression models.

	McFadden	McFadden, Adjusted	Nagelkerke *R*^2^	Veall-Zimmermann	McKelvey-Zavoina
Models assessing predictive value of the variables toward therapy discontinuation (Table 5).					
Assessment 1, *N* = 128.	0.03	−0.06	0.04	0.05	0.05
Assessment 2, *N* = 102.	0.05	−0.18	0.07	0.08	0.15
Exploratory variables, *N* = 128.	0.08	0.00	0.13	0.16	0.15
Models assessing predictive value of the variables toward relapse (Table 6).					
Assessment 1, *N* = 128.	0.09	0.03	0.16	0.20	0.17
Assessment 2, *N* = 102.	0.10	0.01	0.17	0.21	0.18
Assessment 3, *N* = 83.	0.06	−0.06	0.10	0.12	0.10
Exploratory variables, *N* = 128.	0.26	0.19	0.41	0.46	0.47

## Data Availability

Complete raw data are not publicly available due to local data protection laws, but de-identified data can be made available upon reasonable request from the corresponding authors.
